# Different LED light intensity and quality change perennial ryegrass (*Lolium perenne* L.) physiological and growth responses and water and energy consumption

**DOI:** 10.3389/fpls.2023.1160100

**Published:** 2023-04-04

**Authors:** Cátia Brito, Helena Ferreira, Lia-Tânia Dinis, Henrique Trindade, David Marques, Carlos Manuel Correia, José Moutinho-Pereira

**Affiliations:** ^1^ Centre for the Research and Technology of Agro-Environmental and Biological Sciences, University of Trás-os-Montes and Alto Douro, Vila Real, Portugal; ^2^ Institute for Innovation, Capacity Building and Sustainability of Agri-Food Production, University of Trás-os-Montes and Alto Douro, Vila Real, Portugal; ^3^ LOKI, unipessoal Lda., Águeda, Portugal

**Keywords:** energy efficiency, light-emitting diode (LED), light intensity, light quality, perennial ryegrass improvement, photosynthesis, biomass

## Abstract

Light intensity and spectral composition highly affect plant physiology, growth, and development. According to growing conditions, each species and/or cultivar has an optimum light intensity to drive photosynthesis, and different light spectra trigger photosynthetic responses and regulate plant development differently. For the maintenance of natural sports pitches, namely professional football competitions, turf quality is a key condition. Due to the architecture of most football stadiums, the lawns receive low intensities of natural light, so supplementary artificial lighting above the turf is required. The use of light-emitting diodes (LEDs) can have a higher cost–benefit ratio than traditional high-pressure sodium lamps. The continuous emission spectrum, combined with high spectral selectivity and adjustable optical power, can be used to optimize plant growth and development. Thus, perennial ryegrass (*Lolium perenne* L.) plants, commonly used for lawns, were primarily grown at three different intensities (200, 300, and 400 μmol m^−2^ s^−1^) of cool white light. Despite the higher water and energy consumption, 400 μmol m^−2^ s^−1^ maximizes the plant’s efficiency, with higher photosynthetic rates and foliar pigment concentration, and more foliar soluble sugars and aboveground biomass accumulation. Then, it was evaluated the perennial ryegrass (Double and Capri cultivars) response to different spectral compositions [100% cool white (W), 80% Red:20% Blue (R80:B20), 90% Red:10% Blue (R90:B10), and 65% Red:15% Green:20% Blue (R65:G15:B20)] at 400 μmol m^−2^ s^−1^. Both cultivars exhibited similar responses to light treatments. In general, W contributed to the better photosynthetic performance and R90:B10 to the worst one. Water consumption and aboveground biomass were equal in all light treatments. R80:B20 allows energy savings of 24.3% in relation to the W treatment, showing a good compromise between physiological performance and energy consumption.

## 1 Introduction

Light is one of the most important environmental factors, highly affecting plant growth and development (e.g., photosynthesis and photomorphogenesis). The wavelengths of radiation important for environmental plant physiology lie between 300 nm and 1,000 µm, including some of the ultraviolet (UV), the photosynthetically active radiation (PAR), and the infrared (IR) (Taiz and Zeiger, 2006). Light intensity and quality are the main factors that affect photosynthesis, the process by which atmospheric CO_2_ is converted into carbohydrates, which provide energy for all plant functions and structures (Shafiq et al., 2021). Photosynthetically active radiation (between 400 and 700 nm, which is broadly like the visible) represents critical energy for photosynthesis in plants (Wimalasekera, 2019; Shafiq et al., 2021).

Light intensity determines the rate of photosynthesis. At low intensities, but above the light compensation point, the photosynthetic rate increases proportionally to the light intensity, up to a maximum, the light saturation point; furthermore, photosynthetic machinery gets damaged, resulting in photoinhibition (Wimalasekera, 2019). Light quality triggers different responses in plants (Singh et al., 2015). Light in the red (R) region (600–700 nm) resulted in the highest quantum yield of CO_2_ assimilation by plants, followed by the green (G) region (500–600 nm) and then the blue (B) region (400–500 nm) (McCree, 1971). However, plants do not absorb all wavelengths of light equally, with R and B radiation being more efficiently absorbed by photosynthetic pigments than other spectral regions, while a very low amount of G light is absorbed (Olle and Viršilė, 2013; Singh et al., 2015; Liu and van Iersel, 2021). On the other side, the color penetrates differently in the leaf, since B and R light are strongly absorbed within the first 20% of the leaf tissue, while G penetrates deeper and is more uniformly distributed throughout the tissues (Brodersen and Vogelmann, 2010; Liu and van Iersel, 2021), decreasing the potentially negative effect of the internal light gradient within the leaf (Ouzounis et al., 2015). G light can also better penetrate the plant canopy and, thus, potentially increase plant growth by increasing photosynthesis from the leaves in the lower canopy (Kim et al., 2004a).

Apart from the direct effect on photosynthesis, light imparts environmental information, controlling plant development and cellular metabolism, i.e., photomorphogenesis, by being perceived by photoreceptor systems that are mostly activated by R and B light (Taiz and Zeiger, 2006). Red light is important for the development of the photosynthetic apparatus and influences plant morphogenesis by inducing changes in the equilibrium between the inactive and active forms of phytochromes (Taiz and Zeiger, 2006). Blue light, perceived by cryptochromes, is involved in the regulation of various plant processes such as phototropism, inhibition of stem elongation, stomatal opening, gene activation, pigment biosynthesis, and chloroplast movement within the cells (Taiz and Zeiger, 2006).

It is known that R light alone is sufficient for plant growth and photosynthesis (Olle and Viršilė, 2013). However, plants cannot optimally develop with monochromatic R light, and the combination of R and B light has been reported to be favorable for the growth and physiological responses of several crops (Matsuda et al., 2004; Ajdanian et al., 2019; Camejo et al., 2020; Li et al., 2020). At the same time, although normal growth could be achieved with only R and B photons, a proper amount of G light could offer some benefits to the plants, as increases in dry mass accumulation and plant growth have been observed (Kim et al., 2004a; Kim et al., 2004b). On the other hand, G light might function similarly to far-red light and inform the plant of photosynthetically unfavorable conditions and trigger adaptative responses (Folta and Maruhnich, 2007).

The maintenance of natural sports pitches, namely for professional football competitions, has highly competitive and technical requirements. The quality of turf is a key condition not only for the practice of the sport, which is highly regulated, but also for the physical integrity of the players and for the quality of television broadcasts. Raising the quality standards of natural turf and adopting optimized forms of maintenance (reducing consumption and associated costs) are critical factors for the development of the sector and a necessity for sports societies in the management of their infrastructures. Due to the architecture of most football stadiums, which favors the comfort of spectators, the lawns receive low intensities of natural light, and supplementary artificial lighting is required. The supplementary light source is usually provided by high-pressure sodium lamps (HPS) through a car consisting of a main beam with coupled lamps, supported by posts, and moved along the lawn on wheels. However, this illumination has some limitations: it primarily emits yellow and orange but only ~5% of B; it has high radiant and high PAR emission; it has high power consumption; and it has a short life (Astolfi et al., 2012; Terfa et al., 2013). As another option, the use of light-emitting diode (LED) technology has come to stand out as a source of radiation for plant growth in controlled agricultural environments (Olle and Viršilė, 2013; Singh et al., 2015), where G light can be used to facilitate the greenhouse operator’s view in environments with only red/blue light. The continuous emission spectrum, combined with the high spectral selectivity and adjustable optical power of this technology, can be used to optimize plant growth and development. Moreover, it is safe for the user and the environment, has high energy efficiency, low maintenance costs and longevity, leading to great economic savings (Olle and Viršilė, 2013; Singh et al., 2015).

However, the response of plants to different spectral radiations and intensities differs in various species and depends on the growing conditions and plant growth stages (Shafiq et al., 2021). Perennial ryegrass (*Lolium perenne* L.), namely the cultivars developed specifically for turf, is one of the grasses most used for lawns, parks, sports fields, and golf courses (Van Huylenbroeck et al., 1999; OUS, 2022). Several characteristics make this species ideal for lawns because it is quick to germinate, resists the damage caused by use, and recovers quickly from wear. Also, it is persistent and fine-textured (small leaves and tillers), forms dense, uniform, and dark-green turfs, and works well alone and in mixtures with others (Rogers and Lush, 1989; OUS, 2022). Nevertheless, poor shade tolerance is still one of the biggest weaknesses of ryegrass turf-type cultivars. The best new cultivars are still thin and weak in heavy shade and tend to be rapidly replaced by *Poa* and *Agrostis* species and be severely infested with moss (OUS, 2022). In addition, common morphological and physiological responses to low irradiance are frequently observed in plants, such as increased leaf area and decreased leaf thickness, reduced stomatal density and conductance, reduced electron transfer from photosystem II to I and then, lower CO_2_ assimilation rate (Azcón-Bieto et al., 2013; Shafiq et al., 2021). In addition to light intensity, light quality might also affect perennial ryegrass growth, as observed by Barre et al. (2010) in ten *L. perenne* genotypes. In general, shade increased leaf length, and for the same PAR, a decrease in the red/far-red ratio increased leaf elongation rate, and the presence of blue light increased both leaf elongation rate and duration. Still, little is known about the growth and development of perennial ryegrass under LED lighting and the influence of different spectral compositions. This is possibly because this technology is mostly used for plant cultivation under controlled environmental conditions.

The objective of the present study was to optimize the LED lighting spectrum and intensity to properly ensure *L. perenne* growth while improving energy efficiency to be suitably used in sports arenas. As R and B light are believed to be more efficiently absorbed by leaves and together can support normal plant growth, we hypothesized that their combination at an appropriate intensity can be effective in supporting perennial ryegrass growth and allow energy cost savings. To test this hypothesis, two different experiments were conducted under controlled environmental conditions: 1) one aimed to evaluate the *L. perenne* responses to different light intensities and 2) another aimed to evaluate the responses of two common turf-type cultivars (Double and Capri) to different spectral compositions. For both experiments, plant metabolic, physiological, and growth responses, as well as energy consumption were evaluated.

## 2 Material and methods

### 2.1 Plant material and growing conditions

The experiments were conducted from January to April of 2022 at the University of Trás-os-Montes and Alto Douro, Vila Real, Portugal. Perennial ryegrass (*L. perenne* L.) cvs. Double and Capri (Lote DK20HS13252-DGAV and DK21HS0797-DGAV, respectively, Alipio Dias & Irmão, LDA., Portugal) were grown under fully controlled environmental conditions in a growth chamber (215 × 215 × 240 cm). A temperature of 20 ± 1/10 ± 1°C day/night, relative air humidity of 65 ± 5%, and a photoperiod of 14 h light/10 h dark were maintained. The chamber was equipped with LED lamps (OSRAM), each one placed on the top of separate cells (60 × 90 × 72 cm) in a fixed position, ensuring the most homogenous light distribution on a horizontal surface.

The seeds were sown in trays (21.5 × 21.5 × 10 cm) filled with a mixture of substrate (Nutrofertil—Nutrição e Fertilizantes, Lda, Portugal)/perlite (Perligran Premium, Knauf Aquapanel GmbH, Dortmund) (80/20, 800 g) at a density of 0.0173 g cm^−2^ for Double and 0.0151 g cm^−2^ for Capri Cvs. Six days after sowing was considered the time zero, when the seedlings were uniformly emerged. The commercial growing substrate used had the following characteristics: total N 100–150 mg L^−1^, total P (P_2_O_5_) 100–150 mg L^−1^, total K (K_2_O) 120–170 mg L^−1^, organic matter >80%, pH 5.5–6.5. After time zero, irrigation was provided every two days. The trays were watered until field capacity with 150 ml (0.5 ml L^−1^) of a nutritive solution (Complesal 12-4-6, Bayer Crop Science, Portugal) and the remaining with distilled water. The nutritive solution had the following characteristics: N 12%, P_2_O_5_ 4%, K_2_O 6%, B 0.02%, Cu 0.01%, Fe 0.02%, Mn 0.01%, Mo 0.005%, and Zn 0.005%.

### 2.2 Light treatments and experimental design

The work comprised two experiments with the same plant material and growth conditions previously described. Different light treatments were applied to each experiment. The light treatments were set from sowing until the harvest. The spectra of the resulting lamp systems were measured with a spectrometer (StellarNet BLACK-Comet Model CXR-SR, StellarNet Inc., USA). The software SpectraWiz (StellarNet Inc.) was used to acquire and process the data from the sensor.

#### 2.2.1 Experiment 1

**Figure 1:**
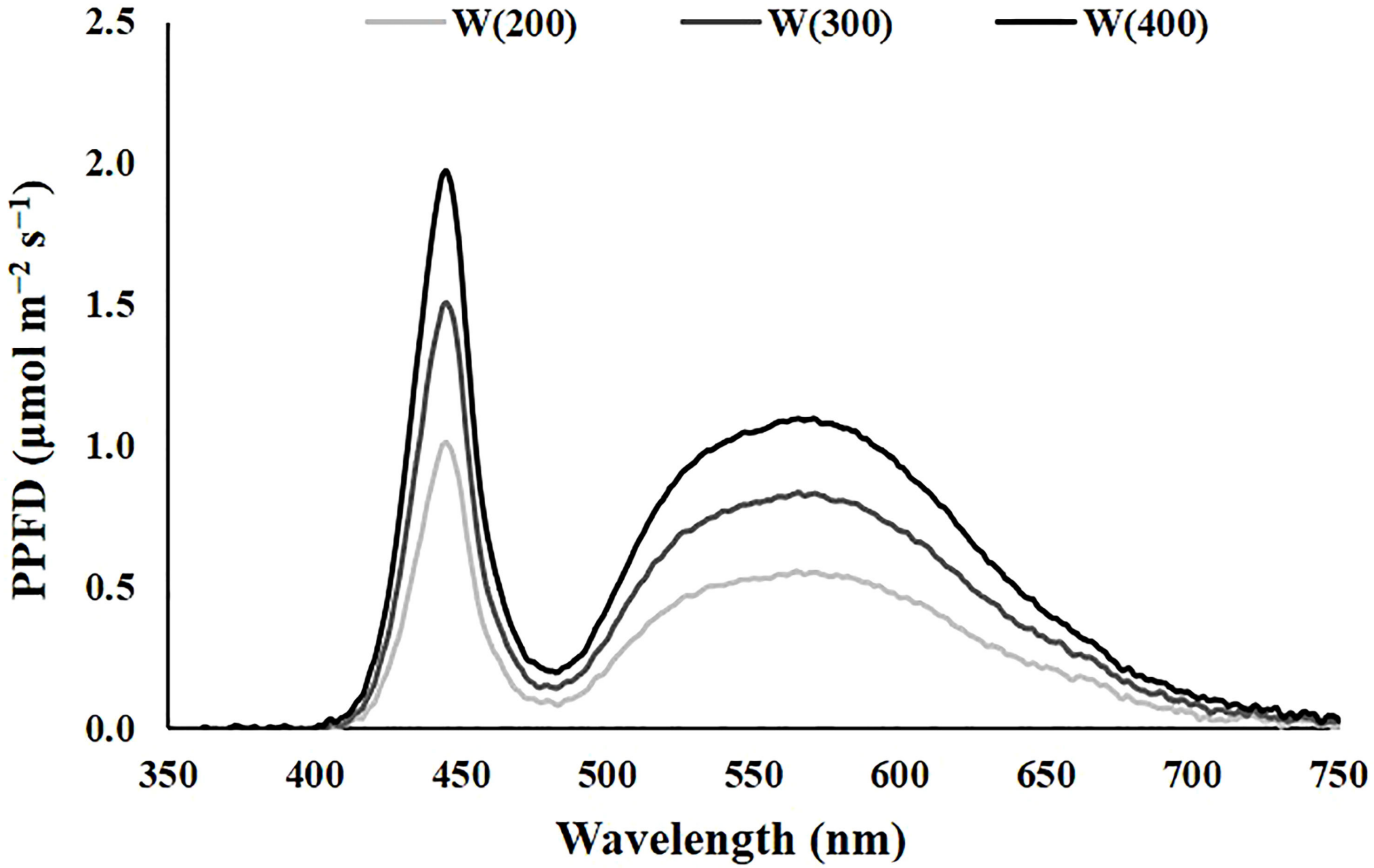
The spectrum of the light treatments applied in experiment 1. Cool white at 200 [W(200)], 300 [W(300)], and 400 [W(400)] μmol m^−2^ s^−1^.

**Figure 2:**
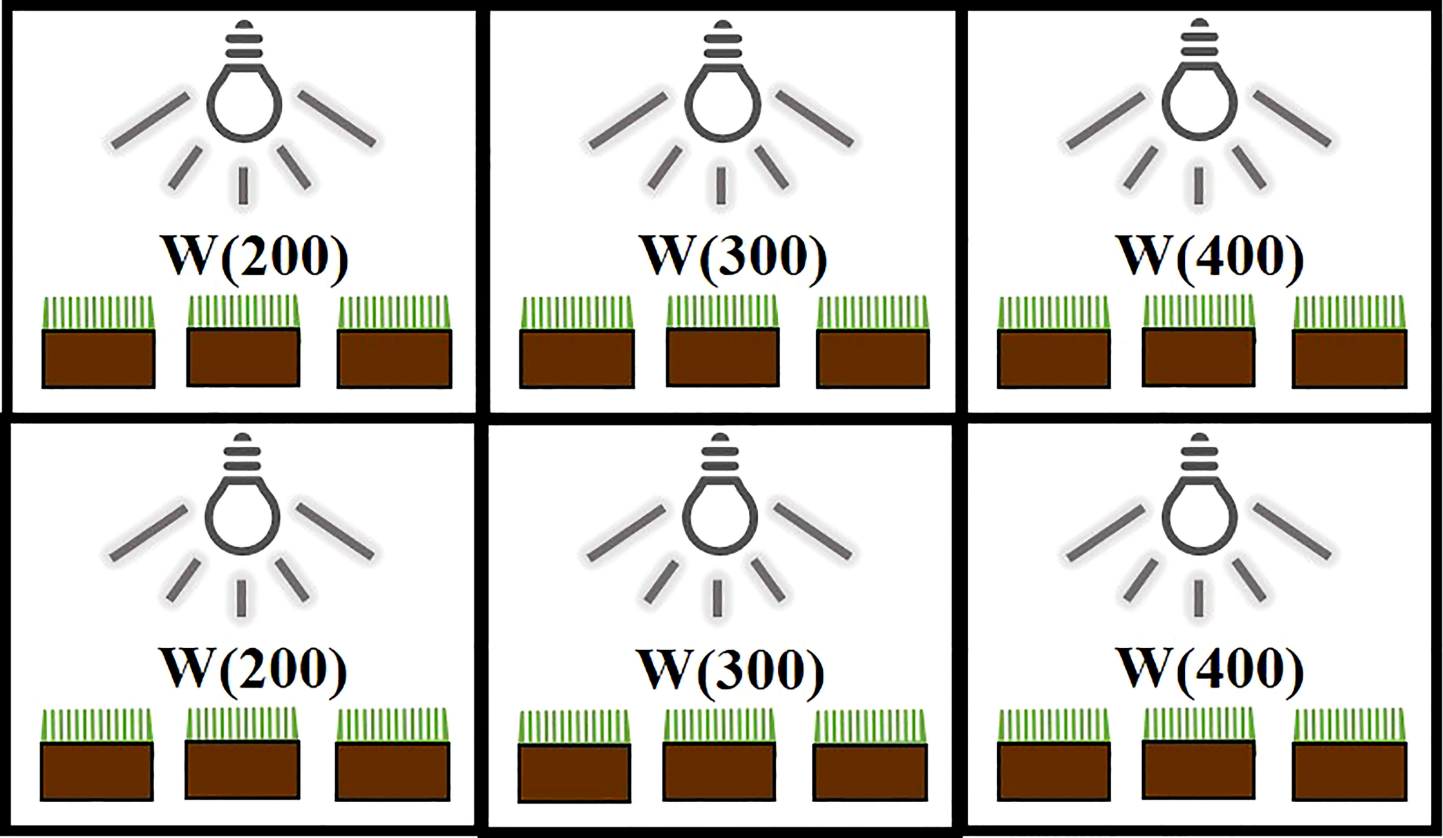
Schematic representation of the experiment 1. Cool white at 200 [W(200)], 300 [W(300)], and 400 [W(400)] μmol m^−2^ s^−1^.

The experiment 1 was performed with cv. Double for 15 days. This preliminary experience aimed to understand the perennial ryegrass response to artificial light and identify a light intensity that allowed a good compromise between energy consumption, physiological performance, and aboveground dry-matter production. To achieve this, three different intensities of cool white light, the broadest spectrum of our system, were tested. The characteristics of the light treatments applied are presented in **Table 1**, and the representation of the spectrum is in **Figure 1**. For each light treatment, six trays of perennial ryegrass were grown, three for physiological and biochemical measurements, and three for biomass accumulation and water consumption evaluation (**Figure 2**). Although uniformity of light distribution in each cell was well achieved, the trays were moved and rotated every two days.

**Table 1:** Proprieties of the treatments applied in experiment 1.

Treatments	PPFD_T_ (μmol m^−2^ s^−1^)	Light spectral ratios	DLI (mol m^−2^ day^−1^)
W (200)	200	24%R: 47%G: 29%B	10.08
W (300)	300	24%R: 47%G: 29%B	15.12
W (400)	400	24%R: 47%G: 29%B	20.16

Total photosynthetic photon flux density (PPFD_T_), light spectral ratios and daylight integral (DLI). Cool white light (W).

#### 2.2.2 Experiment 2

**Figure 3:**
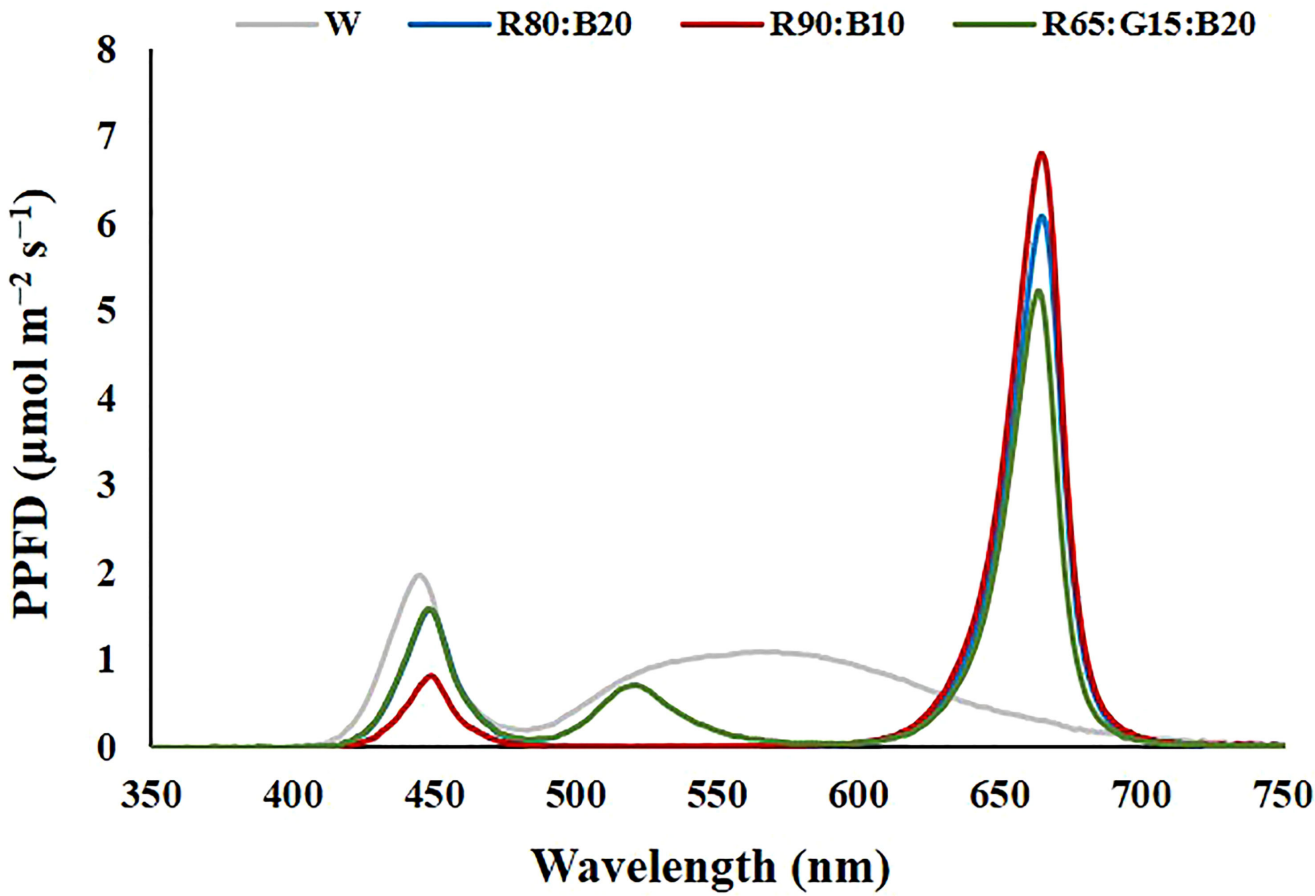
Spectrum of the light treatments applied in experiment 2. 100% cool white (W), 80% Red:20% Blue (R80:B20), 90% Red:10% Blue (R90:B10) and 65% Red:15% Green:20% Blue (R65:G15:B20).

**Figure 4:**
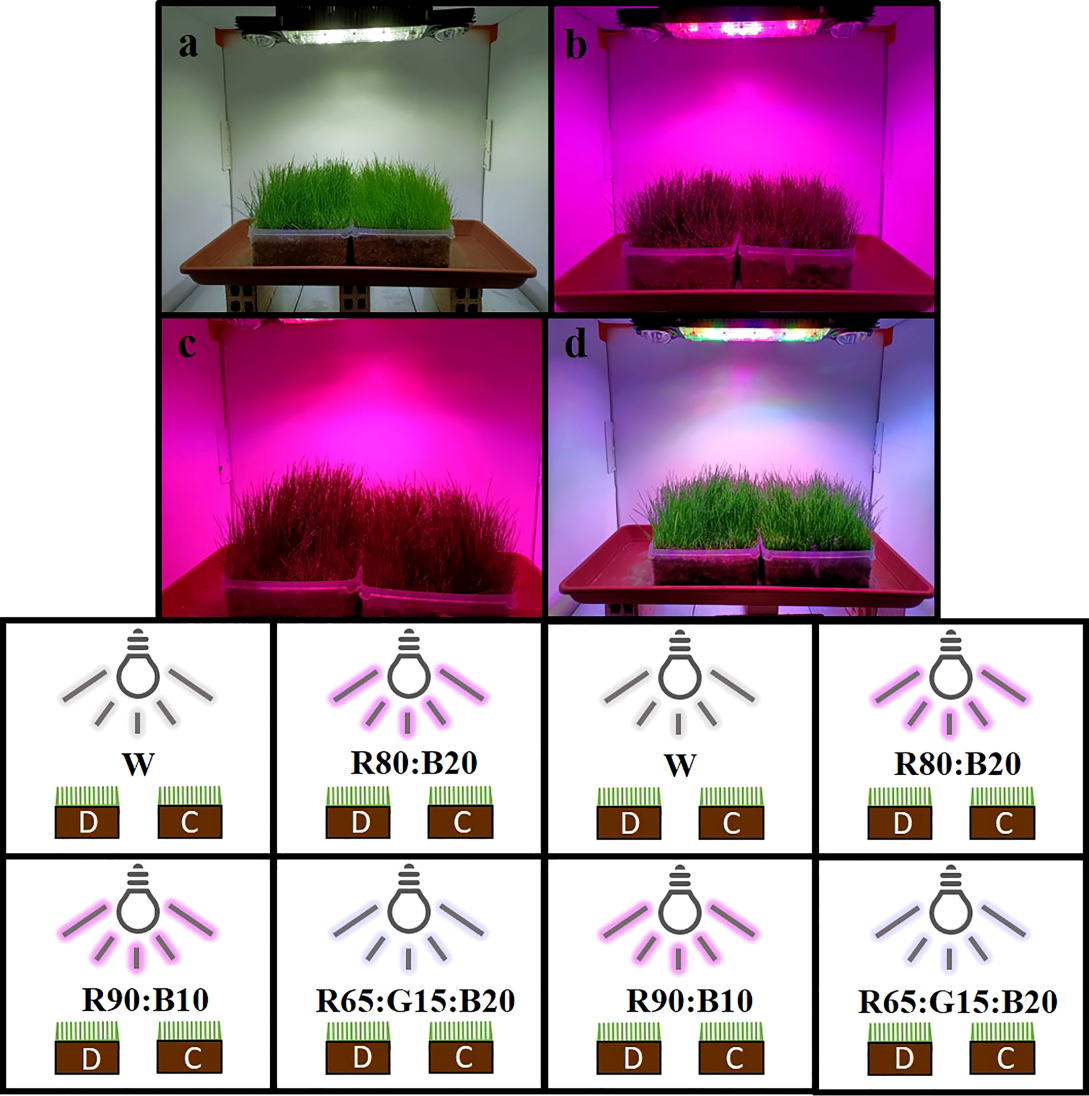
Schematic representation of the experiment 2. **(A)** 100% cool white (W); **(B)** 80% Red:20% Blue (R80:B20); **(C)** 90% Red:10% Blue (R90:B10); and **(D)** 65% Red:15% Green:20% Blue (R65:G15:B20) on Double **(D)** and Capri **(C)** cultivars.

As not enough cells in the light chamber were available to ensure repetitions of four light treatments and two cultivars (Double and Capri), experiment 2 comprises the mean of three different 26-day trials, under the same controlled conditions. The characteristics of the light treatments applied are presented in **Table 2**, and the representation of the spectrum is in **Figure 3**. In each trial, for each light treatment, two trays of perennial ryegrass, cv. Double and cv. Capri, were grown, one for physiological and biochemical measurements and the other for biomass accumulation and water consumption evaluation (**Figure 4**). Although the uniformity of light distribution in each cell was well achieved, the trays were moved and rotated every two days. The grass on the trays selected for physiological and biochemical analysis was cut at 5 cm 8 days after time zero to simulate natural maintenance in sports arenas.

**Table 2:** Properties of the treatments applied in experiment 2.

Treatments	PPFD_T_ (μmol m^−2^ s^−1^)	Light spectral ratios	DLI (mol m^−2^ day^−1^)
**W**	400	24%R: 47%G: 29%B	20.16
**R80:B20**	400	80%R: 20%B	20.16
**R90:B10**	400	90%R: 10%B	20.16
**R65:G15:B20**	400	65%R: 15%G: 20%B	20.16

Total photosynthetic photon flux density (PPFD_T_), light spectral ratios and daylight integral (DLI). Blue (B), red (R) and green (G) light.

### 2.3 Plant measurements

#### 2.3.1 Leaf gas exchange

Leaf gas exchange measurements were performed using a portable IRGA (LCpro+, ADC, Hoddesdon, UK), operating in the open mode. Measurements were performed on a set of leaves uniformly distributed by the IRGA chamber under the corresponding light treatment irradiance. Net photosynthetic rate (A, µmol CO_2_ g^–1^ s^–1^), stomatal conductance (g_s_, mmol H_2_O g^–1^ s^–1^), transpiration rate (E, mmol H_2_O g^–1^ s^–1^) and the ratio of intercellular to atmospheric CO_2_ concentration (C_i_/C_a_) were estimated using the equations developed by von Caemmerer and Farquhar (1981). Intrinsic water use efficiency was calculated as the ratio of A/g_s_ (µmol mol^–1^). Six replicates per selected tray in each trial were performed in two periods, one just before the plants be cut at 5 cm and the other after allowing plants to regrowth for 13 days, because in sport arenas the plants are continuously cut to maintain the desirable carpet conditions, which imposes stress on the plants and can affect their performance. The variables were expressed on a dry mass basis.

#### 2.3.2 Biochemical measurements

At the end of the experiment, per selected tray in each trial, a set of aerial parts of the plants from three zones of the tray was collected (a composite); each one is a biological replication. The composites were reduced to a powder with the help of liquid nitrogen and a mortar and pestle, then stored at −80°C until analysis. From each composite, three technical replications were done for each biochemical analysis, and the results were expressed on a fresh mass basis. Chlorophylls and carotenoids were extracted with acetone/water (80/20, v/v). Chlorophyll a (Chl_a_), chlorophyll b (Chl_b_), and total chlorophyll (Chl_(a + b)_) were determined according to Arnon (1949) and Sesták et al. (1971), and total carotenoids (Car) according to Lichtenthaler (1987). Total soluble sugars (TSS) were extracted according to Irigoyen et al. (1992) by heating the samples in ethanol/water (80/20, v/v) for 1 h at 80°C, and concentration was determined by the anthrone method.

#### 2.3.3 Water consumption and biomass accumulation

The trays selected were weighted every two days after time zero, and water loss was calculated. The same trays were used to evaluate the dry weight of the total aboveground part of the plants. The drying of the plant material was done at 65°C until constant weight.

### 2.4 Energy consumption

The hourly energy consumption of each light regime was measured with a power consumption meter. Data obtained were expressed in kJ per hour.

### 2.5 Statistical analysis

Statistical calculations were performed using the software program SPSS for Windows (v. 22). After testing for ANOVA assumptions (homogeneity of variance with the Levene’s mean test and normality with the Kolmogorov–Smirnov test), statistical differences were evaluated by one-way analysis of variance (ANOVA), followed by the *post-hoc* Duncan’s test (P<0.05).

## 3 Results

### 3.1 Experiment 1

**Figure 5:**
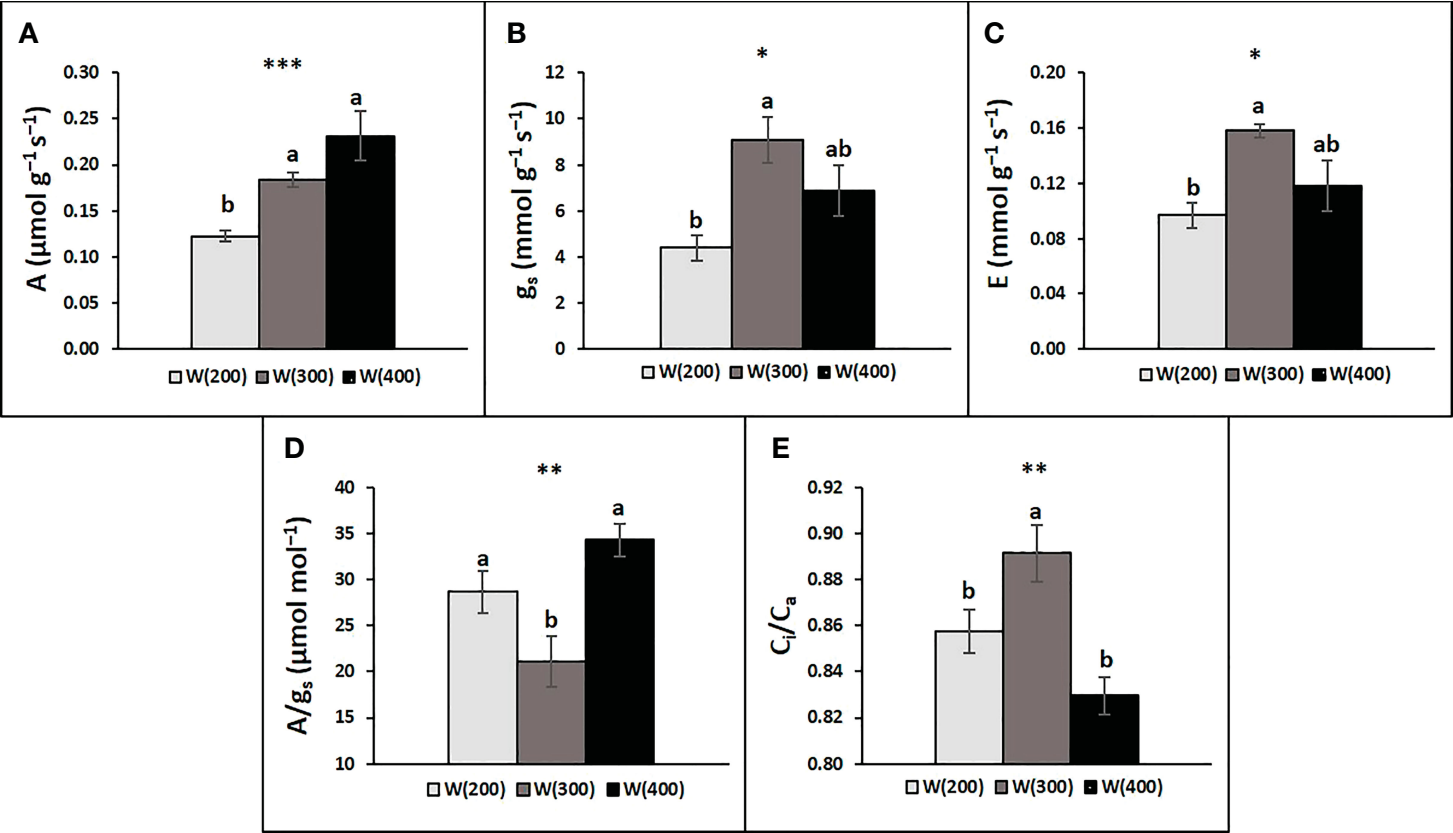
Leaf gas exchange responses of perennial ryegrass (cv. Double) to the different LED illumination regimes, 100% cool white at 200 [W(200)], 300 [W(300)] and 400 [W(400)] μmol m^−2^ s^−1^. Net photosynthetic rate **(A**, a**)**, stomatal conductance **(**g_s_, **B)**, transpiration rate **(E, C)**, intrinsic water use efficiency (A/g_s_, **D**), and ratio of intercellular to atmospheric CO_2_ concentration (C_i_/C_a_, **E**). Each column is average and vertical bars represent the S.E. (n = 18). Different letters indicate significant differences among the treatments (*p<0.05; **p<0.01; ***p<0.001).

The leaf gas exchange analysis demonstrated that light intensity changes perennial ryegrass’ photosynthetic response (**Figure 5A**). A general increase was observed from the 200 to the 400 μmol m^−2^ s^−1^ intensity, although the W(300) did not differ statistically from the W(400). Although both W(300) and W(400) displayed higher g_s_ and E, a statistically significant difference was only observed between W(200) and W(300) (**Figures 5B, C** **)**. Regarding A/g_s_, W(200) and W(400) presented higher values than W(300), while the opposite trend was observed for C_i_/C_a_ (**Figures 5D, E** **)**.

Differences in light intensity also induced changes in foliar photosynthetic pigments and TSS concentrations (**Table 3**). The 200 and 400 μmol m^−2^ s^−1^ intensities induced a higher accumulation of Chl_(a + b)_. On the other hand, no significant differences were recorded between Car and the analyzed ratios, Chl_a_/Chl_b_ and Chl_(a + b)_/Car. The W(400) induced a higher accumulation of TSS.

**Table 3:** Foliar photosynthetic pigments concentration (mg mg^−1^ FW), total chlorophyll (Chl_(a + b)_), chlorophyll a/b ratio (Chl_a_/Chl_b_), total carotenoids (CAR) and Chl_(a + b)_/Car ratio, and total soluble sugars concentration (TSS, mg g^−1^ FW) of perennial ryegrass (cv. Double) under different LED illumination regimes, 100% cool white at 200 [W(200)], 300 [W(300)], and 400 [W(400)] μmol m^−2^ s^−1^.

	Chl_(a + b)_	Chl_a_/Chl_b_	Car	Chl_(a + b)_/Car	TSS
**W (200)**	1.95 ± 0.06^a^	2.81 ± 0.35	0.258 ± 0.028	7.81 ± 1.12	17.4 ± 2.5^b^
**W (300)**	1.73 ± 0.07^b^	3.85 ± 0.78	0.296 ± 0.034	5.99 ± 0.74	20.9 ± 0.8^b^
**W (400)**	1.94 ± 0.08^a^	3.46 ± 0.03	0.336 ± 0.012	5.79 ± 0.10	27.3 ± 0.6^a^
**Significance**	*	ns	ns	ns	**

Values are means ± S.E. (n = 9). Different letters indicate significant differences among the treatments (ns, not significant; *p<0.05; **p<0.01).

**Figure 6:**
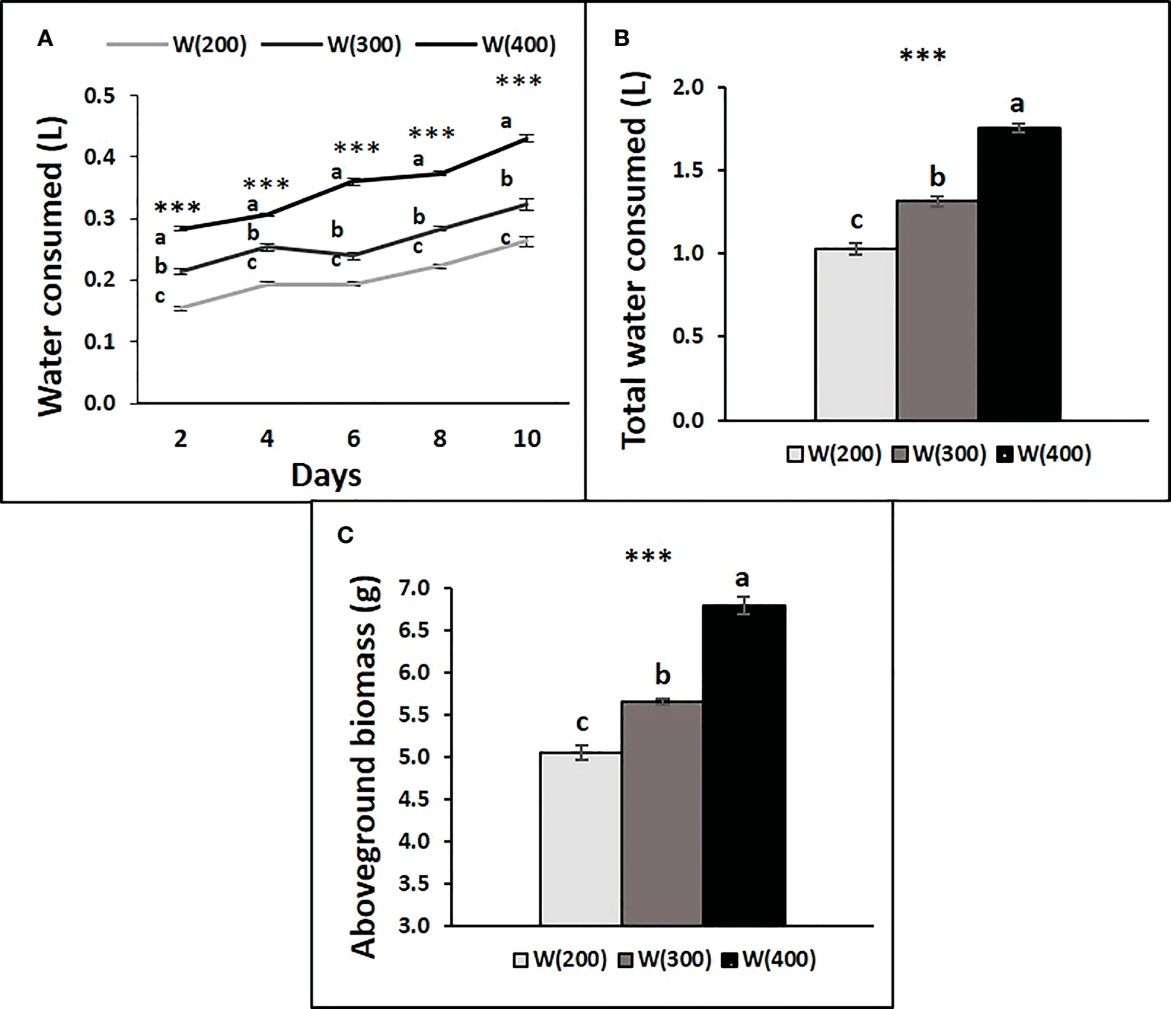
Water consumption by tray of perennial ryegrass (cv. Double) after time zero, every two days **(A)**, accumulated at the end of the experiment **(B)**, and aboveground biomass accumulated by tray at the end of the experiment (g DW, **C**), under different LED illumination regimes, 100% cool white at 200 [W(200)], 300 [W(300)] and 400 [W(400)] μmol m^−2^ s^−1^. Each point and column are average and vertical bars represent the S.E. (n = 3). Different letters indicate significant differences among the treatments (***, p<0.001).

Water consumption by the trays under each light treatment increased across the experimental period (**Figure 6A**). Water was consumed every two days, and the total at the end of the experiment showed an increase from the application of 200 to 400 μmol m^−2^ s^−1^ of W light (**Figures 6A, B** **)**. The accumulation of aboveground biomass followed the same tendency (**Figure 6C**).

### 3.2 Experiment 2

**Figure 7:**
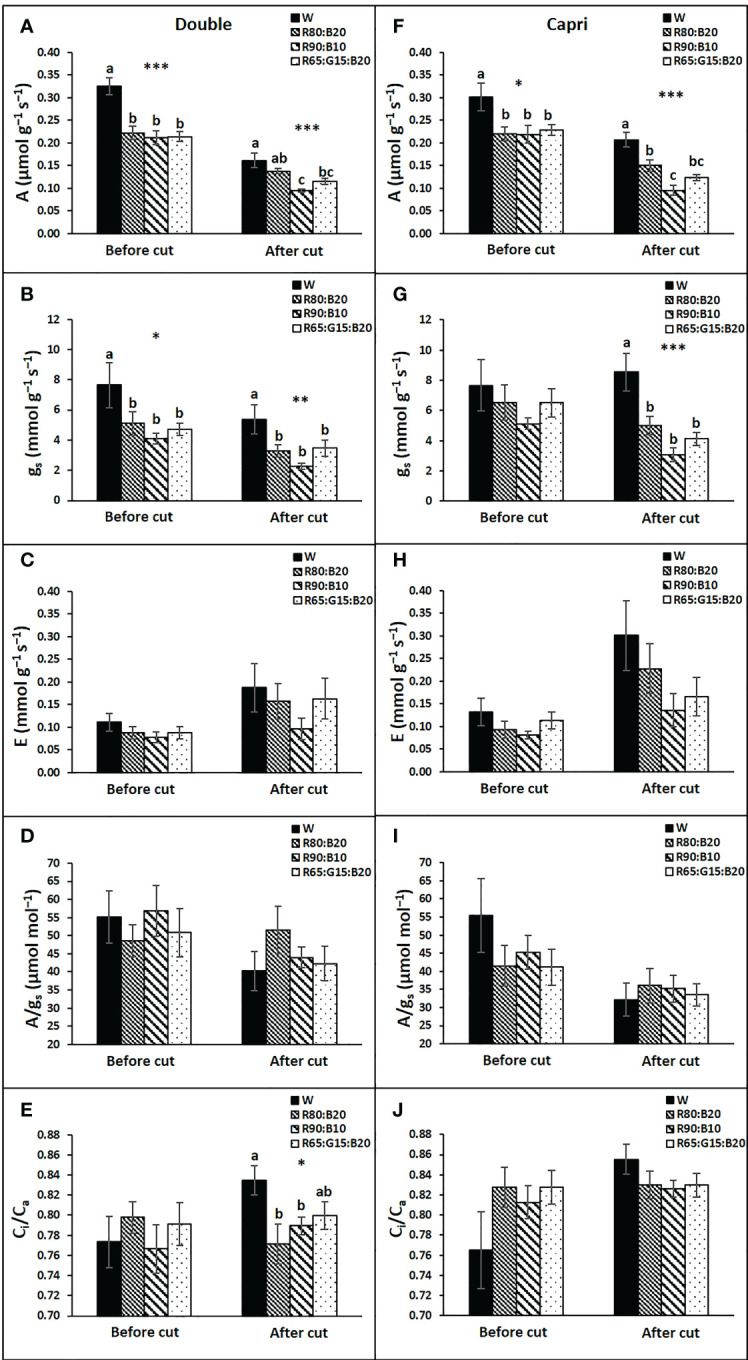
Leaf gas exchange responses of perennial ryegrass (cvs. Double, **A–E**; cv. Capri, **F–J**) to the different LED illumination regimes at 400 μmol m^−2^ s^−1^, 100% cool white (W), 80% Red:20% Blue (R80:B20), 90% Red:10% Blue (R90:B10) and 65% Red:15% Green:20% Blue (R65:G15:B20). Net photosynthetic rate (A, **A** and **F**), stomatal conductance (g_s_, **B** and **G**), transpiration rate **(E, C, H)**, intrinsic water use efficiency (A/g_s_, **D**, **I**) and ratio of intercellular to atmospheric CO_2_ concentration (C_i_/C_a_, **E**, **J**). Each column is average and vertical bars represent the S.E. (n = 18). Different letters indicate significant differences among the treatments (*, p<0.05; **p<0.01; ***p<0.001).

In general, light treatments had similar effects on the gas exchange variables of both cultivars (**Figure 7**). Before cutting the perennial ryegrass, W light potentiated A and g_s_ of both cultivars, although for g_s_ a significant difference was only recorded in the Double genotype (**Figures 7A, B, F, G** **)**. After the cut, a general decrease in A was observed in all treatments, but W continued to have a beneficial effect on A in both cultivars, although in Double, R80:B20 illumination was able to maintain it at the same level as W. In contrast, after cutting, R65:G15:B20 and especially R90:B10 were unfavorable for A (**Figures 7A, F** **)**. The influence of light treatments on g_s_ of both cultivars after the cut was less evident; only W stood out from the other light treatments (**Figures 7B, G** **)**. No significant influence of light treatments was observed on E (**Figures 6C, H** **)** or in A/g_s_ (**Figures 7D, I** **)**. On the other side, in Double, after cutting, R80:B20 and 90:10 decreased the C_i_/C_a_ ratio in relation to W (**Figures 7E, J** **)**.

Light treatments induced changes in foliar photosynthetic pigments and TSS concentrations in a cultivar-dependent manner (**Table 4**). Double showed a higher accumulation of both Chl_(a + b)_ and Car under both W and R80:B20 treatments, while R90:B10 contributed to the lowest Chl_(a + b)_ and Car concentrations. The W and 80:20 treatments contributed to high Chl_(a + b)_/Car ratios: being that, the W stood out. In Capri, W also contributed to a higher concentration of Chl_(a + b)_, but only statistically significant in relation to the R90:B10 treatment, which also induced a lower accumulation of Car in relation to the other treatments. Total soluble sugar concentration was tendentially lower in W and higher in R90:B10 treatments in both cultivars. However, the following sequence can be observed for Double: R90:B10/R80:B20 ≥ R65:G15:B20 ≥ W; and for Capri: R90:B10/R65:G15:B20 ≥ R80:B20 ≥ W.

**Table 4:** Foliar photosynthetic pigments concentration (mg mg^−1^ FW), total chlorophyll (Chl_(a + b)_), chlorophyll a/b ratio (Chl_a_/Chl_b_), total carotenoids (CAR) and Chl_(a + b)_/Car, and total soluble sugars concentration (TSS, mg g^−1^ FW) of perennial ryegrass (cv. Double and Capri) under different LED illumination regimes at 400 μmol m^−2^ s^−1^, 100% cool white (W), 80% Red:20% Blue (R80:B20), 90% Red:10% Blue (R90:B10) and 65% Red:15% Green:20% Blue (R65:G15:B20).

	Chl_(a + b)_	Chl_a_/Chl_b_	Car	Chl_(a + b)_/Car	TSS
Double					
**W**	1.64 ± 0.02^a^	3.33 ± 0.02	0.294 ± 0.007^ab^	5.59 ± 0.09^a^	25.3 ± 1.9^b^
**R80:B20**	1.68 ± 0.03^a^	3.34 ± 0.02	0.309 ± 0.009^a^	5.44 ± 0.11^ab^	31.0 ± 1.0^a^
**R90:B10**	1.33 ± 0.03^c^	3.34 ± 0.03	0.257 ± 0.005^c^	5.19 ± 0.14^b^	33.7 ± 1.6^a^
**R65:G15:B20**	1.46 ± 0.02^b^	3.33 ± 0.04	0.280 ± 0.003^b^	5.21 ± 0.12^b^	30.0 ± 1.7^ab^
**Sig.**	***	ns	***	*	**
Capri					
**W**	1.77 ± 0.08^a^	3.09 ± 0.03	0.305 ± 0.007^a^	5.78 ± 0.13	25.4 ± 1.4^b^
**R80:B20**	1.65 ± 0.07^ab^	3.13 ± 0.03	0.298 ± 0.005^a^	5.51 ± 0.17	30.0 ± 2.6^ab^
**R90:B10**	1.48 ± 0.08^b^	3.11 ± 0.06	0.267 ± 0.006^b^	5.51 ± 0.22	35.5 ± 2.4^a^
**R65:G15:B20**	1.57 ± 0.05^ab^	3.08 ± 0.05	0.289 ± 0.004^a^	5.43 ± 0.20	33.1 ± 2.5^a^
**Sig.**	*	ns	***	ns	*

Values are means ± S.E. (n = 9). Different letters indicate significant differences among the treatments (ns, not significant; *p<0.05; **p<0.01; ***p<0.001).

**Figure 8:**
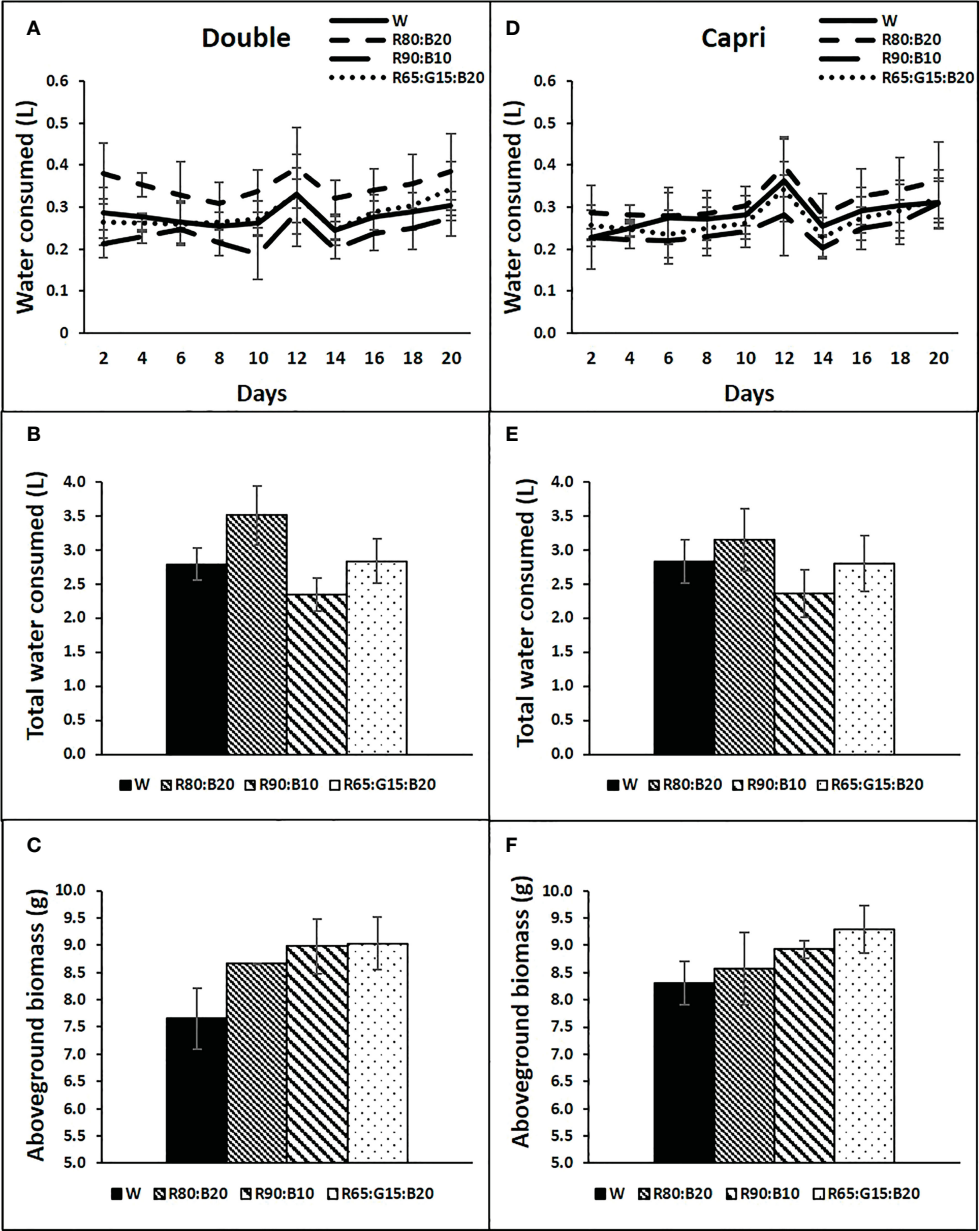
Water consumption by each tray of perennial ryegrass (cvs. Double, **A–C** and Capri, **D–F**) after time zero, every two days **(A, D)**, accumulated at the end of the experiment **(B, E)**, and above ground biomass accumulated in each tray at the end of the experiment (g DW, **C, F**), under different LED illumination regimes at 400 μmol m^−2^ s^−1^, 100% cool white (W), 80% Red:20% Blue (R80:B20), 90% Red:10% Blue (R90:B10) and 65% Red:15% Green:20% Blue (R65:G15:B20). Each point and column are average and vertical bars represent the S.E. (n = 3). The absence of * indicated not statistically differences.

No significant differences were recorded between the applied light treatments and water consumption in any of the cultivars (**Figures 8A, B, D, E** **)**. Water consumption under each light treatment showed a great increase between days 10 and 12 for both cultivars, corresponding approximately 5 days after the ryegrass cut, decreasing again on day 14, and slightly increasing afterwards until the end of the experiment (**Figures 8A, D** **)**. Similarly, light treatments did not significantly affect the aboveground biomass accumulation of both cultivars (**Figures 8C, F** **)**.

### 3.3 Energy consumption

The energy consumption per hour of the evaluated LED regimes is presented in **Table 5**. The energy consumption increases linearly with the increase in light intensity, by 84.7% and 121.8% from 200 to 300 and 400 μmol m^−2^ s^−1^, respectively. Under the intensity of 400 μmol m^−2^ s^−1^, in comparison to 100% cool white [W(400)], an energy saving of 24.3% and 33.2% was observed for R80:B20 and R90:B10, respectively, while the regime R65:G15:B20 increased it by 11.9%.

**Table 5:** Energy consumption (kJ/hour) of the different LED illumination regimes, 100% cool white at 200 [W(200)], 300 [W(300)], and 400 [W(400)] μmol m^−2^ s^−1^ and at 400 μmol m^−2^ s^−1^ 80% Red:20% Blue (R80:B20), 90% Red:10% Blue (R90:B10) and 65% Red:15% Green:20% Blue (R65:G15:B20).

	kJ/h		kJ/h
**W (200)**	146.8	**R80:B20**	246.4
**W (300)**	271.2	**R90:B10**	217.6
**W (4)00**	325.6	**R65:G15:B20**	364.4

## 4 Discussion

### 4.1 Experiment 1

Leaf gas exchange response was evaluated due to the strong relationship between plant productivity and photosynthetic rate (Wimalasekera, 2019). Apparently, the 200 μmol m^−2^ s^−1^ provided by the W LEDs was insufficient to achieve the maximum photosynthetic capacity of perennial ryegrass, as both A and g_s_ were lower. In fact, besides directly affecting light harvesting by plants, as light intensity provides photons carrying energy that excites chlorophyll electrons (Taiz and Zeiger, 2006), light intensity also determines stomatal density, which is higher in higher light environments (Azcón-Bieto et al., 2013), and induces the opening of stomata (Roelfsema and Hedrich, 2005), in order to increase CO_2_ uptake. In line, the higher intensities of light increased g_s_ and A. Although A was slightly higher with W(400) (0.231 µmol $g_{DW}^{-1}$ s^−1^) than with W(300) (0.184 µmol $g_{DW}^{-1}$ s^−1^), no significant differences were recorded between 300 and 400 μmol m^−2^ s^−1^. These results agree with those of Van Huylenbroeck et al. (1999), who evaluated net photosynthesis, at 400 μmol m^−2^ s^−1^, of eleven perennial ryegrass turf commercial cultivars, with average values of 0.240 µmol $g_{DW}^{-1}$ s^−1^. On the other hand, Höglind et al. (2011) found a light saturating PPFD level of about 300 μmol m^−2^ s^−1^ in the range 2–6°C, which increased to about 600 μmol m^−2^ s^−1^ at 9°C. However, the last study was performed with a frozen tolerant cultivar of *L. perenne* (cv. Gunne) used as forage grass.

Chlorophylls *a* and *b* and carotenoids are the most abundant photosynthetic pigments that absorb light and drive the power for photosynthesis. Carotenoids, acting as accessory pigments and absorbing and transferring light to chlorophylls, protect chloroplast structure and function from the damage caused by excessive light (Taiz and Zeiger, 2006; Wimalasekera, 2019). As a rule, when the light intensity is low, the light received is weak, and therefore the plant will produce more photosynthetic pigments (Azcón-Bieto et al., 2013). In fact, W(200) presented a higher Chl_(a + b)_ concentration than W(300). Unexpectedly, W(400) light treatments were able to increase Chl_(a + b)_ concentration at W(200) level. A possible explanation could be the higher capacity of those plants to canalize more photoassimilates to produce more photosynthetic pigments. As no other abiotic factors are limiting the photosynthetic responses, W(400) can be able to catch more light energy.

Usually, under low light intensities, plants invest more in Chl_b_ than in Chl_a_ to take better advantage of the scarce incident radiation, resulting in a reduced Chl_a/b_ ratio (Azcón-Bieto et al., 2013; Wimalasekera, 2019). However, although W(200) plants exhibited the lowest ratio, the results were not statistically significant.

Despite the absence of significant differences in A between W(300) and W(400), W(400) showed higher TSS concentrations in leaves. Moreover, at the end of this experiment, the aboveground biomass accumulation followed a crescent trend with light intensity, increasing 33.4% from W(300) to W(400). This means that the not significant slight increase in A of 400 μmol m^−2^ s^−1^ might have accumulated over the days, resulting in high biomass accumulation and, consequently, higher water consumption. For football pitches, it is important to maintain a dense turf, so the W(400) can have a greater cost–benefit than the W(300).

### 4.2 Experiment 2

Stomatal conductance regulates gas exchange (CO_2_ and H_2_O), and, as a rule, an increase in CO_2_ uptake enhances photosynthesis. A close relation between A and g_s_ is also observed in our results, where the W LEDs highly contributed to higher g_s_ and thus higher A (**Figure 7**). Among all the applied treatments, W is the one with the highest B light percentage (29%), which is known to induce stomatal opening as a signal by being perceived by the phototropins in guard cells and activating the H^+^ -ATPase activity (Inoue and Kinoshita, 2017). However, in general, no significant effects were observed between g_s_ of both R80:B20 and R65:G15:B20 and R90:B10, possibly because stomatal opening in response to weak B light requires background R light, having a synergistic effect with B light response in guard cells (Shimazaki et al., 2007). Red light drives photosynthesis in mesophyll and guard cell chloroplasts and decreases the intercellular CO_2_ concentration (C_i_), which, might induce stomatal opening by a combination of the guard cell response to the C_i_ decrease and a direct response of the guard cell chloroplasts to R light (Shimazaki et al., 2007).

A positive effect of B light on enhancing chlorophyll and carotenoid biosynthesis has also been reported, especially when B light is present at other wavelengths and increases with the given amount of B light (Snowden et al., 2016; Bartucca et al., 2020; Moradi et al., 2021). In line, R90:B10 was the light treatment that contributed to lower Chl_(a + b)_ and Car concentrations, reducing the light absorbance capacity of the plants. These facts help to justify the higher A of R80:B20 and R65:G15:B20 in relation to R90:B10, despite the statistically equal g_s_, and indicate that 10% of B light might not be enough for efficient photosynthetic performance in this species.

In photosynthetic terms, we also observed a close behavior between R80:B20 and R65:G15:B20. Although it is known that R light leads to a higher quantum yield of CO_2_ assimilation than G light (McCree, 1971), some G light characteristics can possibly offset the lower percentage of R light. The G light penetrates deeper in the leaf and is more uniformly distributed throughout the tissues (Brodersen and Vogelmann, 2010); thus, any additional G light absorbed by the lower chloroplasts would increase leaf photosynthesis to a greater extent than would additional R or B light (Terashima et al., 2009), and in some species the G light-absorbing pigment can efficiently channel excitation energy to chlorophyll *a* (Sepúlveda-Ugarte et al., 2011).

Interestingly, although W light increased A, the SS concentration in the aerial part of the plants was lower, suggesting a possible higher exportation to other plant organs such as roots. In line, even though the effect was not statistically significant, the lowest aboveground biomass accumulation tended to occur in the W treatment, which has a great G light percentage (47%). Folta and Maruhnich (2007) suggested that G light could be perceived by the plants as a signal of unfavorable conditions triggering adaptative responses, leading us to believe that the plants under W light could be channeling the photoassimilates to belowground reserve organs. In line, Folta and Maruhnich (2007) concluded in their review that adding G light to the full spectrum resulted in shorter and less fresh/dry mass plants.

## 5 Conclusions

The overall analysis of the experiment leads us to conclude that, despite the higher water and energy consumption, the use of 400 μmol m^−2^ s^−1^ fits better with perennial ryegrass requirements and maximizes dry biomass accumulation.

In experiment 2, despite slight differences, it was observed that both cultivars exhibited similar responses to the applied light treatments. In general, W contributed to better photosynthetic performance and R90:B10 to the worst. Still, water consumption and aboveground biomass were equal in all the experiment 2 treatments, with the W treatment tending to show lower aboveground biomass. Considering the energy consumption data, R80:B20 showed a good compromise between physiological performance and energy consumption, as it allowed savings of 24.3% compared to the W treatment. The interpretation of the results also leads us to conclude that B light has a great influence on the stomatal conductance of this species and that 10% might be a too small amount. Despite G light can has some benefits in photosynthetic response, it is possible that in excess or at the expense of R and B light might compromises the aboveground biomass accumulation.

In sum, 400 μmol m^−2^ s^−1^ of 80% Red and 20% Blue might be a good solution to implement in football stadiums, where the additional natural light can bring a component of G and far-red light, important for plant development.

## Data availability statement

The raw data supporting the conclusions of this article will be made available by the authors, without undue reservation.

## Author contributions

CB and JM-P contributed to conception and design of the study. CB, JM-P, and HF carried out the experiments. CB performed the data analysis and wrote the original draft of the manuscript. DM oversaw the illumination system development. L-TD, HT, and CC revised the manuscript. All authors listed have made a substantial, direct, and intellectual contribution to the work and approved it for publication.
